# Speech Comprehension and Its Relation to Other Auditory Parameters in Elderly Patients With Tinnitus

**DOI:** 10.3389/fnagi.2019.00219

**Published:** 2019-08-21

**Authors:** Zbyněk Bureš, Oliver Profant, Veronika Svobodová, Diana Tóthová, Václav Vencovský, Josef Syka

**Affiliations:** ^1^Department of Auditory Neuroscience, Institute of Experimental Medicine of the Czech Academy of Sciences, Prague, Czechia; ^2^Department of Technical Studies, College of Polytechnics, Jihlava, Czechia; ^3^Department of Otorhinolaryngology of Faculty Hospital Královské Vinohrady and 3rd Faculty of Medicine, Charles University, Prague, Czechia; ^4^Department of Otorhinolaryngology and Head and Neck Surgery, 1st Faculty of Medicine, Charles University in Prague, University Hospital Motol, Prague, Czechia; ^5^Department of Radioelectronics, Faculty of Electrical Engineering, Czech Technical University in Prague, Prague, Czechia

**Keywords:** speech recognition, aging, hearing, auditory temporal processing, tinnitus

## Abstract

Deteriorated speech comprehension is a common manifestation of the age-related decline of auditory functions (presbycusis). It could be assumed that when presbycusis is accompanied by tinnitus, general hearing functions, and particularly comprehension of speech in quiet and speech in noise (SIN), will be significantly affected. In this study, speech comprehension ability and other parameters of auditory function were assessed in elderly subjects with (T, *n* = 25) and without (NT, *n* = 26) tinnitus, aiming for examination of both peripheral and central auditory processing. Apart from high-frequency audiograms in quiet and in background noise, speech recognition thresholds in silence or in competitive babble noise, and the ability to understand temporally gated speech (GS), we measured also sensitivity to frequency modulation (FM) and interaural delay, gap detection thresholds (GDT), or the difference limens of intensity. The results show that in elderly participants matched by age (mean ages around 68 years), cognitive status (median MoCA scores around 27), and hearing thresholds [median pure-tone averages (PTA) around 16 dB hearing loss (HL)], tinnitus *per se* has little influence on speech comprehension. The tinnitus patients also show similar GDT, sensitivity to interaural intensity difference, and sensitivity to FM as the NT subjects. Despite these similarities, nevertheless, significant differences in auditory processing have been found in the tinnitus participants: a worse ability to detect tones in noise, a higher sensitivity to intensity changes, and a higher sensitivity to interaural time differences. Additional correlation analyses further revealed that speech comprehension in the T subjects is dependent on the sensitivity to temporal modulation and interaural time delay (ITD), while these correlations are weak and non-significant in the NT subjects. Therefore, despite similarities in average speech comprehension and several other parameters of auditory function, elderly people with tinnitus exhibit different auditory processing, particularly at a suprathreshold level. The results also suggest that speech comprehension ability of elderly tinnitus patients relies more on temporal features of the sound stimuli, especially under difficult conditions, compared to elderly people without tinnitus.

## Introduction

Tinnitus is the perception of sound in the absence of an objective acoustic stimulus. Apart from a reduction of the quality of life by the phantom auditory sensation itself, people with tinnitus often suffer from worse speech understanding, especially in noisy or complicated listening conditions (Newman et al., 1994; Vielsmeier et al., 2016). Despite the fact that tinnitus is assumed to be mainly triggered by cochlear damage (Eggermont, 1990; Shore et al., 2016), speech comprehension deficits may also occur in tinnitus patients having normal peripheral hearing (Goldstein and Shulman, 1999), normal auditory spectral and temporal resolution (Moon et al., 2015), and normal otoacoustic emissions and auditory brainstem responses (Gilles et al., 2016). For this reason, suprathreshold hearing functions were investigated in some studies with the aim to put the tinnitus into relation with the so-called hidden hearing loss, i.e., the degeneration of suprathreshold auditory nerve fibers and central deafferentation in the absence of elevated behavioral hearing thresholds (Weisz et al., 2006; Schaette and McAlpine, 2011; Ralli et al., 2019). The observed heterogeneity of situations suggest that the reasons for deteriorated speech recognition in tinnitus patients are rather complex; therefore the involvement of both peripheral and central auditory processing and also attentional and cognitive processes have been proposed (Vielsmeier et al., 2016). Previous studies have shown that tinnitus affects working memory and attention (Dornhoffer et al., 2006; Rossiter et al., 2006) and that tinnitus sufferers may have impaired overall cognitive performance (Andersson et al., 2000) and auditory stream segregation (Durai et al., 2019).

Speech understanding deficits are frequently emerging as a consequence of aging and age-related hearing loss (presbycusis; Mazelová et al., 2003). Other audiometric parameters, such as pure tone audiograms (Jilek et al., 2014), gap detection thresholds (GDT), intensity discrimination, or sensitivity to temporal features (Grassi and Borella, 2013; Profant et al., 2015, 2019), also change with age. Clearly, the age-related decline in auditory processing will interact with tinnitus, possibly also altering the speech comprehension ability. In general, the literature on speech understanding in people with tinnitus is rather scarce and to our knowledge, there is no study that would explore the joint effect of tinnitus and presbycusis on speech understanding and how this ability correlates with the various measures of auditory processing. At the same time, both presbycusis and tinnitus represent major health and social problems with a large clinical engagement (Roth et al., 2011; McCormack et al., 2016). Therefore, to address this important issue, we specifically analyzed the tinnitus and speech comprehension in elderly subjects (with a mean age of approximately 68 years). First, we tested the hypothesis that tinnitus in the elderly people would worsen their comprehension of speech in quiet and under difficult conditions, by comparing groups of participants with and without tinnitus. In addition, by using an extended battery of auditory tests (Profant et al., 2015, 2019), we aimed for a more profound examination of both peripheral and central auditory processing in these people. The audiometric tests were complemented by the cognitive screening and subjective evaluation of tinnitus, using Tinnitus Handicap Inventory (THI) questionnaire.

As shown by previous studies, simple comparisons of average values are often not sufficient and more information could be obtained by using correlation analysis (Moon et al., 2015; Riedl et al., 2015; Profant et al., 2019). Therefore, as the next step, the categorical analysis was complemented with correlation analysis to find possible differences in trends and relationships (correlations) between speech understanding and various audiometric factors.

## Materials and Methods

### Participants

Fifty-one participants were examined in this study: 25 subjects (mean age ± SEM: 66.84 ± 0.3 years; 14 women and 11 men) with tinnitus (T), and 26 subjects (mean age ± SEM: 68.92 ± 0.2 years; 13 women and 13 men) without tinnitus (NT). All of the examined participants declared no previous otologic surgery, vestibular lesion, severe head trauma, lesion of the facial nerve, disorder of the cervical spine, or self-reported central nervous system disorder. Several of the participants occasionally played musical instruments. An otoscopic examination, with the removal of the cerumen and confirmation of an intact tympanic membrane, was performed on all subjects. The examination procedures were approved by the Ethics Committee of the Motol University Hospital, in Prague. All subjects gave written informed consent in accordance with the Declaration of Helsinki.

### Measurement and Stimulation

All the measurements were performed in a sound-attenuated audiometric booth. Acoustic signals were delivered *via* high-frequency audiometric headphones, Sennheiser HDA 300, connected to a custom-made audiometric apparatus based on a high-quality audio device (RME Fireface), complemented by a custom-made programmable attenuator. The apparatus provided a digital-to-analog conversion and attenuation/amplification of the measurement signals, communication between the experimenter and the examined subject, and an acquisition of the subject’s responses using a comfortable interface with backlit buttons (Arturia BeatStep). The apparatus was controlled by a custom-made software package built in the Matlab environment, which provided all the necessary functions including the generation and/or playback of digital measurement signals, acquisition of the subjects’ responses, and basic data evaluation. The equipment was calibrated according to ISO 389-5, ISO 389-8, ISO 8253-3 and IEC 60645-3 standards using the Brüel and Kjær 4153 Artificial Ear. The following audiometric parameters were assessed:

- high-frequency pure-tone audiograms in quiet,
- pure-tone audiograms in background noise,
- speech recognition thresholds in silence speech recognition score (SRS),
- speech recognition thresholds in competitive babble noise speech in noise (SIN),
- recognition thresholds of temporally gated speech (GS),
- sensitivity to interaural time delay (ITD) and interaural level difference (ILD),
- detection thresholds of frequency modulation (FM),
- detection thresholds of gap in white noise (GDT),
- difference limen for intensity (DLI) of a tone in noise (DLI_tone_) and of white noise bursts (DLI_noise_).

Pure-tone audiograms in quiet were obtained in an extended frequency range from 125 Hz to 16 kHz (0.125, 0.25, 0.5, 0.71, 1, 1.6, 2, 3.15, 4, 6.3, 8, 10, 12.5, and 16 kHz, starting from the lowest frequency), separately for each ear (starting with the left ear). The hearing thresholds were measured automatically using the following procedure: at a given frequency, the stimulation started at 50 dB HL. In cases when the subject did not respond, the intensity was increased in 20 dB steps until either a response or the maximum allowed intensity was reached. When the subject started to respond, the intensity was decreased in 4 dB steps as long as the subject kept on responding; afterward, the intensity was increased in 2 dB steps until the subject’s response was again obtained. This up-down procedure was repeated until the subject responded twice at the same intensity. The percentage of positive responses was plotted as a function of intensity; the function was fitted with a sigmoidal Boltzmann-like function to obtain a parameterized approximation of the psychometric function. The threshold was estimated as the intensity of the fit at the mid-point. Only subjects with no significant difference between the left-ear and right-ear hearing thresholds were included in the study so that the thresholds of both ears could be evaluated together. The pure tone average (PTA) was given as a mean hearing loss (dB HL) at 0.5, 1, and 2 kHz. Furthermore, a wide-range pure-tone average (PTAw) was calculated from all the measured frequencies up to 10 kHz.

To test basic suprathreshold auditory performance, pure-tone audiograms in white noise background were measured at 0.5, 1, 2, 3, 4, 6, and 8 kHz, and the noise level was set to 60 dB SPL. Pure-tone averages in noise (PTA_noise_, and PTAw_noise_) were calculated in a manner similar to PTA and PTAw, respectively.

For the measurements of speech comprehension, all the signals (speech, and the background noise if appropriate), were presented simultaneously to both ears. The speech audiometry in quiet utilized a standard set of Czech word audiometry according to Seeman (1960). At each measured intensity, 10 words were presented and the SRS (percentage of understood words) was registered. The threshold was set to the intensity where SRS = 50%. The speech audiometry in babble noise speech in noise (SIN) employed a standard set of Czech sentence audiometry, according to Dlouhá et al. (2012). The speech level was always kept at 65 dB SPL and the background babble noise level was increased in 3 dB steps starting at 63 dB SPL. For each noise level, 10 sentences were delivered and the recognition score (percent of sentences understood) was registered. The recognition score was plotted as the function of the noise level, and the noise level corresponding to the 50% recognition score was taken as the threshold. In addition, periodically gated speech (GS) was measured: short sentences (see the speech audiometry in noise) were periodically gated (cycle duration 200 ms, 15 ms raised cosine ramps) with a given duty cycle (approximately 30% to 60%); the percentage represents the proportion of the total cycle duration containing the speech signal, the remaining segment of the cycle was muted. The measurement procedure was analogous to that of SIN. The measurement proceeded in 10% steps from small (usually 30%) to larger values of duty cycle until the recognition score reached nearly 100%. The recognition score was plotted as the function of the duty cycle, and the duty cycle corresponding to the 50% recognition score was taken as the threshold.

Sensitivity to ITD and ILD was extracted from a more complex measurement of a binaural time-intensity interchange ratio (Profant et al., 2019). The subjects were exposed to click trains (10 clicks, 100 μs duration; 10 Hz repetition rate; SPL = 100 dB, measured as peak-to-peak equivalent SPL, calibrated according to EN ISO 60645-3) with different interaural time and intensity differences. The time-intensity trading completely or partly compensates one parameter (which simulates lateralization to one side) by the other parameter (which simulates lateralization to the other side), resulting in the perception of the signal in the middle or lateralized position, respectively, which is indicated by the subject. The responses were analyzed by means of non-linear surface fitting by cubic smoothing splines, giving a smooth and high-resolution approximation of the subject’s lateralization responses over the whole range of interaural time and intensity differences. The surface fitting was performed in Matlab software (Mathworks Inc., Natick, MA, USA) using CSAPS function (Profant et al., 2019). The sensitivity to ITD (or ILD) was extracted by cutting the interpolated surface with a vertical plane at zero intensity difference (or zero time difference, respectively). To obtain a parameterized approximation of a psychometric function, the resulting cut was fitted using a sigmoidal Boltzmann-like function *b*:

$$b\left(x\right)=\frac{2}{1+e^{\left(\mu -x\right)/\delta }}-1$$

where *x* stands for the independent variable (intensity or time difference), and *μ* and *δ* are estimated parameters. The sensitivity to ITD or ILD was evaluated as the slope (first order derivative) of the corresponding fit in the mid-point.

The sensitivity to FM was measured using a frequency-modulated tone (1 kHz carrier, 60 dB SPL), presented simultaneously to both ears at several modulation frequencies (2, 4, 8, 16, and 32 Hz) with a variable modulation index. Each trial in this test consisted of two successive tones (duration 1 s, interval 200 ms, inter-trial interval 3 s), one of them being frequency modulated by a sinusoidal modulation function. By pressing one of two buttons, the subject had to indicate whether the modulation was present in the first or in the second tone. The test starts with a well audible modulation index (1%). After two successive correct answers at a given modulation index, the modulation index is halved. After an incorrect answer, the modulation index is doubled. This way, the modulation index is repeatedly increased and decreased, resulting in a zigzag character. The measurement of the given modulation frequency finishes after the second turn to lower modulation indexes. The percentage of correct answers was plotted against the modulation index, fitted using a sigmoidal Boltzmann-like function, and the threshold was estimated as the modulation index of the fit in the mid-point. An average value computed from all the modulation frequencies was then taken as the result.

For the measurement of GDT, three successive pauses in a continuous white noise at 70 dB SPL (150 ms intervals between gaps) were presented. Both the noise and the gaps were delivered simultaneously to both ears. The gap duration was varied (usually with 0.1 ms step) until the detection threshold was obtained. The resulting value of the threshold was the gap duration for which the subject’s detection score equaled 50%.

The DLI of tone in noise was assessed using 1 kHz tone at 60 dB SPL embedded in a continuous background white noise at 60 dB SPL; the DLI of white noise was assessed using white noise bursts at 60 dB SPL presented in silence. Each trial in this test consists of two successive stimuli (duration 1 s, interval 200 ms, inter-trial interval 3 s), the first being a reference stimulus with constant intensity, the second one being a test stimulus with randomly chosen intensity difference. By pressing one of two buttons, the subject has to indicate whether the test stimulus has the same or different intensity compared with the reference. Each tested intensity (intensity differences were selected from the range between 0 dB and ±7 dB) is repeated 10 times during the session. The percentage of responses “different” is calculated depending on the intensity difference. The result is typically a V-shaped function with a minimum close to 0 intensity difference. To evaluate the threshold intensity difference, the responses to a given absolute intensity difference (e.g., ±4 dB) are taken together, the percentage of responses “different” is calculated and the result is plotted depending on the absolute intensity difference, obtaining a psychometric function. The liminal intensity difference is determined visually, as the intensity difference where the linearly interpolated dependence crosses 50%.

### The Assessment of Tinnitus Annoyance and Cognitive Abilities

The tinnitus annoyance was determined using a THI questionnaire (Newman et al., 1996, 1998). Cognitive screening was performed using MoCA questionnaire (Nasreddine et al., 2005). This test examines several cognitive domains, in particular, short-term and working memory, visuospatial abilities, attention, concentration, language capability, abstraction, or orientation to time and place. The highest possible MoCA score is 30; scores around 26 are considered as normal at the age of 65, scores around 25 are considered normal at the age of 85 (Borland et al., 2017).

### Statistical Analysis

The normality of distributions of the data sets was tested using the Shapiro-Wilk test prior to subsequent analyses. Due to the fact that many of the data sets were found to have a distribution significantly deviating from normality, two-sided Wilcoxon rank-sum tests were computed for comparisons of the medians of two data sets. In the case of age and hearing thresholds in quiet, the data are presented as mean ± SEM. Otherwise, the data are presented as medians and interquartile ranges in the form median|IQR. The possible relationships between the selected parameters were tested using Spearman’s correlation coefficient. In all cases, the alpha level was set to 0.05. In each group of participants (T and NT), 21 correlations were performed. To control for possible effects of multiple correlations, we estimated an appropriate *p*-value by creating a *p*-value plot (Schweder and Spjøtvoll, 1982; Santurette and Dau, 2012). The outcome suggests that the significance level used in the correlation analysis (*α* = 0.05) is appropriate and does not need correction. In addition, partial Gini indexes were used to verify the correlation analysis (Strobl, 2005; Menze et al., 2009). Statistical analyses were performed using Matlab software (Mathworks Inc., Natick, MA, USA).

## Results

In total, 25 participants with tinnitus (T) and 26 participants without tinnitus (NT) were measured in this study. The mean ages of the experimental groups were matched to be as close as possible (NT: 68.92 ± 0.2 years; T: 66.84 ± 0.3 years) to exclude potential age-related differences. According to the THI questionnaire, most tinnitus patients rated their tinnitus as slight (*n* = 16). Only a minority of T subjects rated their tinnitus as mild (*n* = 5), moderate (*n* = 3), or severe (*n* = 1). Approximately half of the tinnitus subjects reported the tinnitus laterality as central (*n* = 13), less frequent was left (*n* = 7) and right (*n* = 5) laterality.

Cognitive abilities were tested using a MoCA test. Both groups of participants performed almost equally well (NT: 27.5|3.3; T: 27.0|2.0; *p* > 0.05, Wilcoxon rank-sum test), with two subjects in each group scoring 23 or lower.

### The Comparison of Audiometric Results Between the Groups

**Figure 1:**
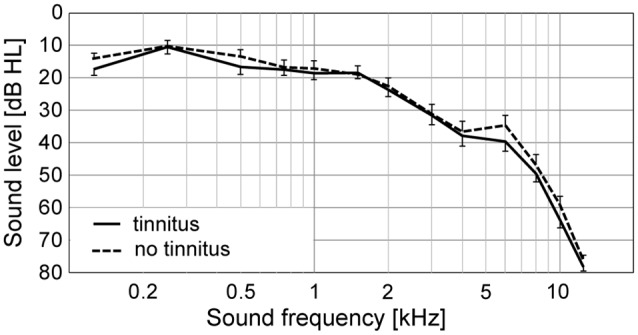
Average pure-tone audiograms of the subjects with (T) and without (NT) tinnitus. Mean ± SEM.

**Figure 2:**
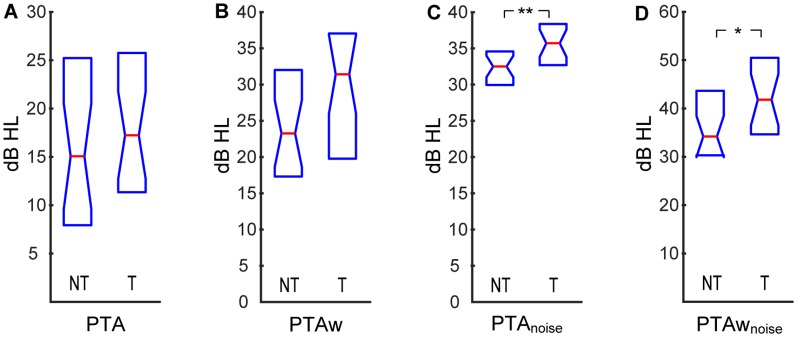
Pure-tone averages (PTA) computed for audiograms in quiet and in white noise: PTA **(A)**, wide-range pure-tone average (PTAw; **B**), PTA in white noise **(C)**, PTAw in white noise **(D)**. Medians and IQR. Statistically significant difference denoted with asterisks (Wilcoxon rank-sum tests; *p < 0.05; **p < 0.01).

At first, we compared the audiometric parameters between the two experimental groups. Average hearing thresholds in quiet of both groups are illustrated in Figure 1. The (PTA and PTAw) did not differ significantly between the tinnitus and control groups (PTA NT: 15.0|16.1 dB HL, T: 17.2|14.0 dB HL; PTAw NT: 23.1|12.7 dB HL, T: 31.3|17.8 dB HL; *p* > 0.05 in both cases, Wilcoxon rank-sum tests, see Figures 2A,B). However, the hearing thresholds of tones in noise were significantly worse in the tinnitus group compared with the control group, see Figures 2C,D (PTA_noise_ NT: 32.4|4.6 dB HL, T: 35.6|4.9 dB HL, *p* < 0.01; PTAw_noise_ NT: 34.1|13.0 dB HL, T: 41.7|14.3 dB HL, *p* < 0.05; Wilcoxon rank-sum tests).

**Figure 3:**
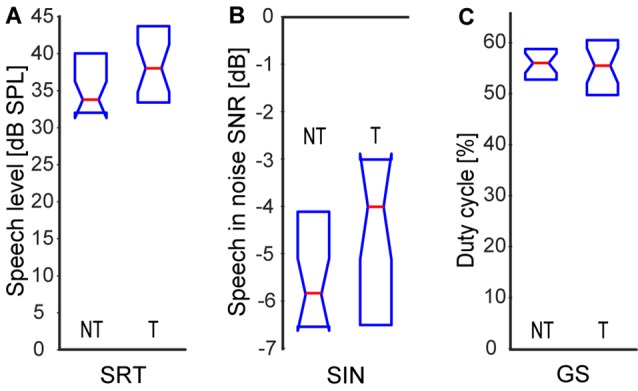
Speech comprehension: speech recognition threshold in quiet **(A)**, speech in babble noise **(B)**, gated speech (GS; **C**). Medians and IQR.

Speech comprehension ability did not show any significant differences between the T and NT groups in any of the three tests, see Figure 3 [SRS NT: 33.5|8.0 dB SPL, T: 38.0|9.4 dB SPL; SIN NT: 70.8|2.4 dB SPL (SNR −5.8|2.4 dB), T: 69.0|3.5 dB SPL (SNR −4.0|3.5 dB); GS NT: 56.0|5.5%, T: 55.5|10.4%; *p* > 0.05 in all cases, Wilcoxon rank-sum tests].

**Figure 4:**
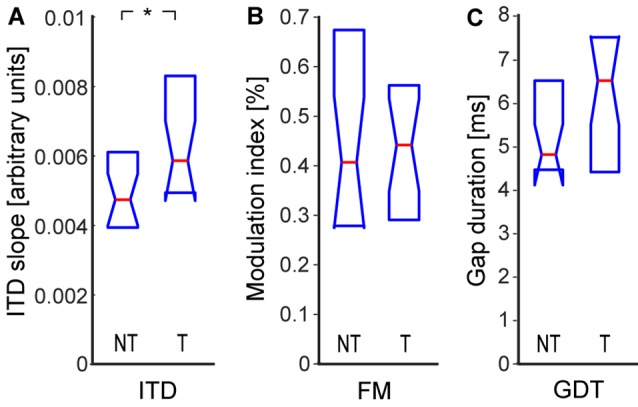
Sensitivity to temporal parameters: interaural time delay (ITD) sensitivity **(A)**, sensitivity to frequency modulation (FM; **B**), gap detection threshold (GDT; **C**). Medians and IQR. Statistically significant difference denoted with asterisk (Wilcoxon rank-sum tests; *p < 0.05).

In this study, we measured three types of parameters that are related to the processing of temporal information carried by the stimulus waveform: sensitivity to ITD, sensitivity to FM, and detection thresholds of short gaps in continuous noise. The sensitivity to interaural delay was significantly enhanced in the tinnitus group (NT: 0.0047|0.0021, T: 0.0059|0.0032, *p* < 0.05; Wilcoxon rank-sum test; Figure 4A). The detection thresholds of FM were not significantly different (NT: 0.39|0.39%, T: 0.44|0.25%, *p* > 0.05; Wilcoxon rank-sum test), see also Figure 4B. Interestingly, the GDT were also not significantly different in the tinnitus and control groups (NT: 4.8|2.0 ms, T: 6.5|2.9 ms, *p* > 0.05; Wilcoxon rank-sum test), see Figure 4C. To test the possibility that our result was due to the fact that most of our tinnitus participants declared slight tinnitus severity in the THI questionnaire, we computed the correlation of the GDT and THI scores: the two quantities were uncorrelated (*ρ* = 0.11, *p* > 0.05, Spearman correlation).

**Figure 5:**
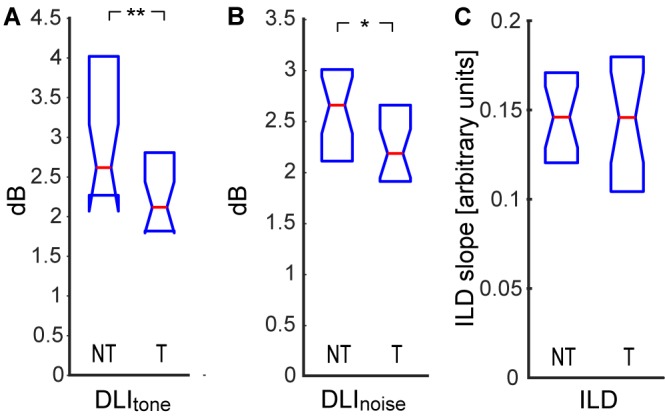
Difference limen for intensity (DLI) of tone in noise **(A)**, DLI of noise **(B)**, sensitivity to interaural level difference (ILD; **C**). Medians and IQR. Statistically significant difference denoted with asterisks (Wilcoxon rank-sum tests; *p < 0.05; **p < 0.01).

To further test suprathreshold auditory performance, we also measured the smallest detectable change in the intensity of a 1 kHz tone in a white noise background (Figure 5A), the smallest detectable change in the intensity of white noise bursts (Figure 5B), and sensitivity to interaural difference in intensity (ILD, Figure 5C). Contrary to our expectations, we found that the tinnitus participants had a smaller DLI in both tests, indicating a higher sensitivity to suprathreshold intensity changes (DLI_tone_ NT: 2.6|1.6 dB, T: 2.1|0.9 dB, *p* < 0.01; DLI_noise_ NT: 2.6|0.9 dB, T: 2.2|0.7 dB, *p* < 0.05; Wilcoxon rank-sum tests). To test if this result could relate to a potentially narrower intensity dynamic range of people with elevated thresholds, we examined the relationship between the DLI and the PTA_noise_ and found no correlation (DLI_tone_: *ρ* = 0.02, *p* > 0.05; DLI_noise_: *ρ* = 0.17, *p* > 0.05; Spearman correlations). The improved performance was also probably not caused by better temporal acuity of the T subjects, as the DLI did not correlate with any of the measures of temporal acuity (DLI_tone_ vs. ITD *ρ* = −0.15; DLI_tone_ vs. FM *ρ* = −0.07; DLI_tone_ vs. GDT *ρ* = −0.05; DLI_noise_ vs. ITD *ρ* = −0.11; DLI_noise_ vs. FM *ρ* = 0.16; DLI_noise_ vs. GDT *ρ* = 0.02; *p* > 0.05 in all cases, Spearman correlations). Interestingly, the sensitivity to ILD was almost equal in both groups (NT: 0.14|0.05, T: 0.15|0.07, *p* > 0.05, Wilcoxon rank sum test).

### The Relationship of Speech Comprehension Ability With Other Parameters

To discover possible relationships between different factors, we performed a correlation analysis of selected audiometric parameters. For the reason that speech comprehension is one of the crucial factors influencing the quality of life of the tinnitus subjects, we particularly examined the relationships of the speech recognition measures with other auditory and non-auditory parameters. The results are summarized in Table 1.

**Table 1:** Correlations of speech comprehension ability with other audiometric parameters in the T and NT groups.

		PTA	PTAw	GDT	FM	ITD	MoCA	THI
T subjects	SRS	*ρ* = 0.90***	*ρ* = 0.74***	*|ρ|* < 0.2^ns^	*ρ* = 0.46*	*|ρ|* < 0.2^ns^	*ρ* = −0.38^ns^	*ρ* = 0.25^ns^
	SIN	*ρ* = 0.62**	*ρ* = 0.85***	*ρ* = 0.45*	*ρ* = −0.46*	*ρ* = 0.31^ns^	*ρ* = 0.27^ns^	*|ρ|* < 0.2^ns^
	GS	*ρ* = 0.70***	*ρ* = 0.84***	*ρ* = 0.56**	*ρ* = 0.47*	*ρ* = −0.44*	*ρ* = −0.25^ns^	*|ρ|* < 0.2^ns^
NT subjects	SRS	*ρ* = 0.84***	*ρ* = 0.84***	*|ρ|* < 0.2^ns^	*ρ* = 0.32^ns^	*|ρ|* < 0.2^ns^	*|ρ|* < 0.2^ns^	N/A
	SIN	*ρ* = 0.57**	*ρ* = 0.77***	*ρ* = 0.61**	*|ρ|* < 0.2^ns^	*ρ* = −0.33^ns^	|*ρ*| = 0.2^ns^	N/A
	GS	*ρ* = 0.77***	*ρ* = 0.87***	*ρ* = 0.58**	*ρ* = 0.22^ns^	*ρ* = 0.27^ns^	*ρ* = −0.23^ns^	N/A

Spearman correlation coefficients (*ρ*) are given along with overall significance of correlation (*p < 0.05, **p < 0.01, ***p < 0.001, ns—not significant). In cases where the absolute value of the correlation coefficient was smaller than 0.2, *|ρ|* < 0.2 is indicated.

At first, it was natural to quantify the relationship between speech recognition and hearing thresholds. Both PTA and PTAw correlated strongly and significantly with all the speech intelligibility measures in both groups. The correlation coefficients of PTA and speech recognition measures (T vs. NT groups; Spearman correlation) were: SRS *ρ* = 0.90 vs. *ρ* = 0.84, *p* < 0.0001 in both cases; SIN *ρ* = −0.62 vs. *ρ* = −0.57, *p* < 0.01 in both cases; GS *ρ* = 0.70 vs. *ρ* = 0.77, *p* < 0.0001 in both cases. The correlation coefficients of PTAw and speech recognition measures were similarly high (T vs. NT groups; SRS *ρ* = 0.74 vs. *ρ* = 0.84, *p* < 0.0001 in both cases; SIN *ρ* = −0.85 vs. *ρ* = −0.77, *p* < 0.0001 in both cases; GS *ρ* = 0.84 vs. *ρ* = 0.87, *p* < 0.0001 in both cases; Spearman correlation).

To examine the influence of temporal parameters on speech comprehension, we computed correlations of GDT, sensitivity to FM, and sensitivity to ITD with speech parameters. The GDT did not correlate with SRS (*ρ* < 0.2, *p* > 0.05 in both groups; Spearman correlation); however, significant correlations of GDT were present in both groups in the case of degraded speech (correlation coefficients T vs. NT: SIN *ρ* = −0.45 vs. *ρ* = −0.61, *p* < 0.05 vs. *p* < 0.01; GS *ρ* = 0.56 vs. *ρ* = 0.58, *p* < 0.01 in both cases). Sensitivity to FM was associated with intelligibility of speech in quiet (SRS) in the people with tinnitus (*ρ* = 0.46, *p* < 0.05; Spearman correlation), but not in the NT group (*ρ* = 0.32, *p* > 0.05; Spearman correlation). Similarly, a higher sensitivity to FM only improved SIN comprehension in the T group (T: *ρ* = −0.46, *p* < 0.05; NT: *ρ* = −0.1, *p* > 0.05; Spearman correlation). This was the same regarding GS (T: *ρ* = 0.47, *p* < 0.05; NT: *ρ* = 0.22, *p* > 0.05; Spearman correlation). Finally, in the tinnitus group, the higher ITD sensitivity enhanced the ability to understand GS (*ρ* = −0.44, *p* < 0.05; Spearman correlation); no such correlation was found in the NT group (*ρ* = 0.27, *p* > 0.05; Spearman correlation). The ITD sensitivity did not correlate with SRS (*ρ* < 0.2, *p* > 0.05 in both groups; Spearman correlation). In the case of SIN comprehension, no significant correlation with ITD was present (T: *ρ* = 0.31, NT: *ρ* = −0.33, *p* > 0.05 in both groups; Spearman correlation), however, the correlation coefficients had the opposite sign (the tinnitus patients showed better speech comprehension ability with higher ITD sensitivity, while the NT subjects behaved in the opposite way). We also, therefore, computed linear regression fits and compared their slopes. The result showed that the slopes were significantly different (linear regression with an *F*-test, *p* < 0.01).

Obviously, speech comprehension may also be dependent on non-auditory factors, such as THI, or cognitive abilities. Cognitive abilities given by the MoCA score were not significantly correlated with speech intelligibility in our participants (*p* > 0.05 in all cases; Spearman correlation), which might be caused by a small variance of the MoCA scores found in the study. In the tinnitus patients, we also examined the dependence of speech comprehension on THI, but we found no significant correlations (*p* > 0.05 in all cases; Spearman correlation). To further check this result, we compared speech comprehension abilities in tinnitus patients with the THI score “slight” (denoted as T1) and those having a THI score worse than “slight” (denoted as T2), but no statistically significant differences were found [T1 vs. T2; SRS: 37.5|9.6 vs. 41.0|13.0 dB SPL; SIN: 69.3|3.1 vs. 68.5|4.1 dB SPL (SNR −4.3|3.1 vs. −3.5|4.1 dB); GS: 55.0|7.9 vs. 58.5|12.3; *p* > 0.05 in all cases; Wilcoxon rank-sum tests]. However, the effect sizes for these tests estimated using Cohen’s *d* were medium-to-high in the case of SRS and GS (SRS *d* = 0.67, GS *d* = 0.75), therefore the tinnitus annoyance might influence speech comprehension; statistical significance could be achieved for a larger population of participants.

### The Comparison of Tinnitus Subjects With Larger THI Scores

Since the THI score could potentially influence the audiometric results, we performed separate comparisons of NT participants with those tinnitus participants, who had declared THI scores worse than “slight” (T2 group). In these analyses, we focused on those parameters where we expected a possible worsening: PTA, PTAw, SRS, SIN, GS, and GDT. However, no significant differences were found even with these more handicapped subjects—see Table 2 (Wilcoxon rank-sum tests). Nevertheless, when looking at the values of Cohen’s *d*, it can be seen that the effect sizes were mostly medium (except in the case of speech in noise, where the effect size is small), thus the differences could reach significance with larger samples and may be potentially important.

**Table 2:** Comparison of NT participants with tinnitus patients having Tinnitus Handicap Inventory (THI) scores worse than “slight” (T2 group).

Parameter	NT (median|IQR)	T2 (median|IQR)	*p*-value (Wilcoxon rank-sum test)	Cohen’s *d*
PTA (dB HL)	15.0|16.1	28.4|21.3	>0.05	0.56
PTAw (dB HL)	23.1|12.7	36.6|18.8	>0.05	0.43
SRS (dB SPL)	33.5|8.0	41.0|13.0	>0.05	0.53
SIN (dB SPL)/	70.8|2.4/	68.5|4.1/	>0.05	0.12
SIN SNR (dB)	−5.8|2.4	−3.5|4.1
GS (%)	56.0|5.5	58.5|12.25	>0.05	0.51
GDT (ms)	4.8|2.0	6.95|1.05	>0.05	0.54

## Discussion

In the current study, we investigated auditory functions in aged humans with and without tinnitus, focusing on the joint effect of presbycusis and tinnitus on speech comprehension ability. Apart from standard pure-tone audiograms (extended to high-frequency region) and speech recognition thresholds in silence, we aimed to test also suprathreshold auditory performance (by measuring pure-tone audiograms in background noise, comprehension of speech in babble noise, and difference limens of sound intensity with and without masking noise), and auditory temporal processing [by measuring sensitivity to interaural delay (ITD) and FM, GDT, and the ability to understand temporally GS]. The measures of auditory function were compared between the T and NT participants, furthermore, a correlation analysis was performed in order to find how speech comprehension abilities relate to auditory functions, cognitive status, and tinnitus annoyance.

The data show that the tinnitus itself may not cause worse speech understanding and that speech comprehension in tinnitus patients is related to pure-tone audiograms in a very similar way as in people without tinnitus. However, contrary to the controls, the tinnitus participants show significant correlations of speech understanding with several metrics of auditory temporal processing, i.e., FM or ITD. Furthermore, the different intensity discrimination and tone-in-noise detection in the T group suggest that suprathreshold auditory processing is affected.

Many audiometric parameters (in particular, pure-tone thresholds, frequency and intensity discrimination ability, gap detection ability, or detection of amplitude modulation) deteriorate with age (Grassi and Borella, 2013; Füllgrabe et al., 2015). The age-related auditory changes are continuous and progress even at higher age; for example, Grassi and Borella (2013) reported numerous differences between groups of subjects with mean ages of 68 and 81 years, Jilek et al. (2014) demonstrated that changes of hearing thresholds are well detectable in groups of subjects differing in mean age by only 5 years. Taking these facts into account and considering that a larger difference in age could substantially bias the results, the two experimental groups were matched in age as closely as possible. The two groups also happened to have very similar hearing thresholds. As the influence of hearing thresholds could also be substantial, particularly for speech comprehension, this fact eliminates another possible source of unwanted variability. The average pure-tone audiograms of both groups correspond well to the reference hearing thresholds of normal-aging human participants of their age (Jilek et al., 2014). Despite normal audiograms, however, the tinnitus subjects exhibit significantly elevated tone detection thresholds when measured with background noise. Very recently, a similar test (tone-in-noise detection) was used by Ralli et al. (2019) to compare suprathreshold auditory performance in young and elderly patients. Our results suggest that the suprathreshold auditory perception is affected differently than near-threshold hearing in the tinnitus group, a result also confirmed by the tests on the smallest detectable change of intensity (DLI, see below).

One of the most important complaints of people with hearing difficulties is that they suffer from poor speech understanding. In this study, we did not exclusively select subjects with subjectively degraded speech understanding—the main criterion for inclusion in the study was the existence of tinnitus, and age. Our data show that speech comprehension in participants with and without tinnitus can be similar, both for speech in quiet and for speech in noise, which is in contrast to other studies that reported worse speech comprehension in people with tinnitus (e.g., Huang et al., 2007; Ryu et al., 2012; Moon et al., 2015; Gilles et al., 2016). This discrepancy is most probably caused by differences in the experimental procedures and in the choice of subjects. A major difference between our study and the cited articles is that we focused specifically on aged people while the cited works used participants with wider age span; it is thus possible that speech comprehension ability is influenced more by aging than by tinnitus, so when both phenomena (presbycusis and tinnitus) coexist, the effect of age dominates and masks the effect induced by tinnitus. To test this possibility, a comparison of tinnitus patients of various ages would be necessary; such a study is planned in our future research activities. Our results on speech comprehension may also be related to the fact that our tinnitus subjects mostly reported only a slight tinnitus annoyance in the THI questionnaire. Despite the lack of statistical significance, supplementary analyses exclusively examining people with a worse than slight THI score (see Table 2) suggest that the subjective severity of the tinnitus might play a role, as the effect sizes (Cohen’s *d*) of majority of the comparisons show medium to high values. The study of Huang et al. (2007) reported, however, that there was no correlation between speech perception and THI score, which is in accordance with our basic results. Similarly, a study of Newman et al. (1994) also found no relationship between speech measures and subjectively perceived handicap. These results show that the situation is more complex, and that to find out which tinnitus characteristics influence speech perception would require a detailed multidimensional characterization of the phantom perception. Overall, our data indicate that it is not possible to draw a general conclusion that people with tinnitus always have worse speech comprehension ability; rather, speech comprehension ability depends on various auditory and non-auditory factors and their interactions. Furthermore, psychological aspects, such as subjective acceptance of tinnitus or attention, could potentially bear importance. According to Riedl et al. (2015), a higher acceptance of tinnitus results in lower psychological distress and better mental health of patients, Brozoski et al. (2019) reported decreased auditory attention in tinnitus subjects—these factors might affect speech understanding *via* non-auditory influences.

In both participant groups, speech comprehension ability is similar and well correlated with pure-tone audiograms in quiet in all the three tests. Interestingly, there is no difference in the SIN understanding, despite that in the corresponding psychophysical task—detection of a signal in noise background—the T subjects performed worse than the NT subjects. This outcome suggests that when no additional pathology is present, the hearing threshold is still the most important predictor of speech understanding performance in the elderly both with and without tinnitus. Nevertheless, it does not mean that speech comprehension does not depend on other factors. In the case of degraded speech (speech in noise and GS), but not speech in silence, the comprehension ability is correlated with GDT in both participant groups, which agrees with previous findings (Tyler et al., 1982; Festen and Plomp, 1983; Gordon-Salant and Fitzgibbons, 1993). Importantly, only the tinnitus subjects are able to benefit in all three speech recognition tests from the sensitivity to temporal modulation; in addition to this, the sensitivity to ITD also enhances speech recognition in the tinnitus subjects in contrast to the NT people. Therefore, it can be assumed that in spite of the similar average performance of both groups in the tests on temporal processing (except the ITD sensitivity, where the T subjects performed better), the temporal information is more important for speech comprehension in people with tinnitus compared to the NT controls. This result agrees with previous observations by Moon et al. (2015), who reported that sensitivity to temporal modulation was significantly correlated with SIN recognition in ears with tinnitus, while this correlation was weaker and non-significant in ears without tinnitus. It may also be that humans with tinnitus can utilize the temporal information more efficiently, perhaps to overcome or compensate the perceptual deficit given by the phantom sensation. Contrary to our findings, a correlation between temporal resolution and speech in noise understanding has been found in non-tinnitus patients by Festen and Plomp (Festen and Plomp, 1983). This difference is probably due to differences in subject groups—in particular, the participants used by Festen and Plomp were markedly younger than those of ours.

The tinnitus subjects performed better in several suprathreshold tests: sensitivity to ITD, DLI of a tone in noise, and DLI of white noise bursts. The improved sensitivity to ITD again suggests a higher importance of temporal features of the stimulus for the tinnitus patients, as mentioned above. A similar finding can be found in Moon et al. (2015): normal hearing subjects with tinnitus exhibited better ability to detect temporal modulation than control subjects without tinnitus. These improvements in temporal processing may have developed in order to compensate for the perceptual deficit resulting from the tinnitus sensation. Another explanation could be that they represent a consequence of an increased neural synchrony and spontaneous rates reported in relation to tinnitus at various levels of the auditory pathway (Bauer et al., 2008; Ahlf et al., 2012; Eggermont and Tass, 2015; Wu et al., 2016). As the increased synchrony can already be observed in the cochlear nucleus, i.e., before the first binaural neurons (Wu et al., 2016), and subsequently also in inferior colliculus (Bauer et al., 2008), it can affect both binaural processing of ITD presented here, and the sensitivity to modulations found by Moon et al. (2015). The improved DLI may perhaps relate to loudness recruitment or, more generally, to an altered shape of rate-intensity functions of suprathreshold neurons in the tinnitus participants. Unfortunately, our current data cannot provide a full explanation of this phenomenon, as we found no significant correlation of DLI with masked thresholds. The improved DLI’s found in our study are in contradiction to the work of Epp et al. (2012); in our opinion, the result of Epp et al. (2012) could have been biased by the large age difference between their subject groups—the tinnitus subjects were on average 12 years older than the controls. In any case, together with the worse masked audiograms observed in the T group, these findings indicate an altered processing of stimuli at suprathreshold levels. In rodents, an altered suprathreshold function of the auditory periphery has recently been identified even in animals with normal hearing thresholds; this phenomenon was attributed to a selective degeneration of synapses at cochlear hair cells and has been known as the “hidden hearing loss” (Kujawa and Liberman, 2015; Rybalko et al., 2015). For human subjects, however, controversy exists in relation to hidden hearing loss. For example, Mehraei et al. (2016) stated that this phenomenon existed in humans and could be detected using the measurement of auditory brainstem responses; on the contrary, recent works of Guest et al. (2017, 2018) failed to link the presumed cochlear synaptopathy with any electrophysiological measure in people both with and without tinnitus. Our data indicate that different suprathreshold auditory processing could develop in tinnitus patients, yet with no direct relation to tone thresholds in quiet or speech comprehension.

It is also an interesting fact that the tinnitus participants have a similar gap-detection ability to the NT group, which is contradictory to the idea that the phantom sensation should mask the gaps in noise (Sanches et al., 2010; Galazyuk and Hébert, 2015). An explanation of our result may be based on the fact that the gaps in our experiments were embedded in broad-band noise; the gap-masking paradigm might work better in the case of narrowband noise tuned to the tinnitus pitch (Fournier and Hébert, 2013; Rybalko et al., 2019) although this possibility is also doubted by some studies (Campolo et al., 2013; Boyen et al., 2015). Another factor is the subjective tinnitus annoyance: our T group consisted mostly of participants with only a slight tinnitus handicap (given by the THI questionnaire) and thus the gap-masking might not be very efficient. The supplementary analysis of subjects with a worse THI score (T2 group) suggests that the subjective severity of tinnitus could play a role—the effect size of the GDT difference between the NT and T2 groups is medium and thus the difference could reach statistical significance with a larger number of observations.

In the current study, tinnitus severity was judged based on the THI questionnaire; psychometric tinnitus assessment (loudness and pitch matching) could not be performed due to limitations on examination time of the patients. It is conceivable that subjective tinnitus loudness might influence some of the obtained results. Unfortunately, there is not an agreement on whether the THI score used in our work may serve as an index of subjective tinnitus loudness: some studies have shown that tinnitus loudness judgments correlate well with self-rated annoyance and THI (e.g., Newman et al., 1994; Degeest et al., 2016), other studies found no correlation between subjective tinnitus ranking or THI and matched loudness (Meikle et al., 1984; Nascimento et al., 2018). Future work is hence needed to fully clarify this issue. Considering the subjective tinnitus pitch, the situation appears to be less unclear. Previous studies have shown that tinnitus pitch is not associated either with tinnitus severity (Meikle et al., 1984; Degeest et al., 2016), or with other audiometric parameters such as audiogram (Pan et al., 2009; Keppler et al., 2017) or GDT (Boyen et al., 2015). It can thus be assumed that tinnitus pitch would have only a little influence on our results. In any case, it appears that various tinnitus descriptors provide outcomes with different mutual relationships and also different relationships to general audiometric parameters; complete understanding of the topic is still lacking.

According to the generally accepted view, tinnitus develops as a consequence of peripheral (inner-ear) damage due to associated plastic changes in the central auditory system (Tan et al., 2013). A common assumption is that tinnitus is an unwanted side-effect of adaptive processes which constantly optimize information transmission and try to compensate for altered input from the periphery by increasing neuronal gain, spontaneous activity, and neuronal synchrony (Bauer et al., 2008; Wu et al., 2016; Krauss et al., 2018). In our results, the assumed increased synchrony may be behind the improved ITD sensitivity. The damage of outer hair cells was suggested to be responsible for tinnitus development (Mitchell and Creedon, 1995), however, more recent works state that inner hair cell dysfunction may serve as the triggering mechanism (Tan et al., 2013; Moon et al., 2015). This opinion is also in line with our results: the unchanged hearing thresholds in the tinnitus patients indicate that no tinnitus-specific outer hair cell damage is present, while the suprathreshold processing is different and points to altered processing of intensity. In this context, it is surprising that SIN comprehension is not affected in the tinnitus participants; perhaps if the SIN test was performed with different speech levels, a certain effect would appear.

The correlation analysis employed 21 correlations in each participant group, bringing up the problem of multiple comparisons. In these situations, corrections for multiple comparisons are usually applied to prevent false rejections of valid null hypotheses. On the other hand, these corrections may lead to not detecting true differences especially due to their low power caused by small sample size. In order to find the balance between these two possible errors and to settle the boundary between null hypotheses to keep and null hypotheses to reject, we created a *p*-value plot (Schweder and Spjøtvoll, 1982; Santurette and Dau, 2012). According to the result, the hypotheses to reject correspond exactly to those in which the *p*-value was smaller than 0.05, thus we consider the significance level *α* = 0.05 appropriate, with no need for correction. Furthermore, the correlation results were confirmed by additional analysis of variables worth, using partial Gini indexes as measures of internal classification impurity (Strobl, 2005; Menze et al., 2009).

In conclusion, this study shows that with matched hearing thresholds, the tinnitus *per se* does not lead to worse speech comprehension ability in aged subjects, neither in quiet nor under difficult conditions. Furthermore, no significant association between tinnitus and poorer gap-detection ability has been found. On the other hand, the tinnitus participants exhibit an increased receptiveness to temporal features of the stimulus. First, the sensitivity to interaural time differences (ITD) is higher than in no-tinnitus participants; furthermore, correlations between measures of auditory temporal processing and speech comprehension ability are stronger in the tinnitus group. The tinnitus patients thus seem to utilize the temporal information in a more efficient manner or, viewed from the opposite angle, temporal information is more important for speech understanding in humans with tinnitus than in people without this handicap. In addition, despite no differences in pure-tone audiograms, the suprathreshold auditory function is affected in the tinnitus patients, as indicated by worse tone-in-noise detection and better intensity discrimination abilities. This fact may relate either to the controversial “hidden hearing loss” or, more generally, to an altered shape of rate-intensity functions of auditory neurons accompanying the plastic changes induced by the tinnitus.

## Data Availability

All datasets generated for this study are included in the manuscript.

## Ethics Statement

The examination procedures were approved by the Ethics Committee of the Motol University Hospital, in Prague. All subjects gave written informed consent in accordance with the Declaration of Helsinki.

## Author Contributions

ZB: manuscript preparation, design of auditory tests, data analysis, data interpretation. OP: design of auditory tests, data interpretation, manuscript preparation. VS, DT, and VV: examination of subjects. JS: manuscript preparation, project overview.

## Conflict of Interest Statement

The authors declare that the research was conducted in the absence of any commercial or financial relationships that could be construed as a potential conflict of interest.

## Abbreviations

DLI_noise_: difference limen for intensity of white noise bursts
DLI_tone_: difference limen for intensity of a tone in noise
FM: frequency modulation
GDT: detection thresholds of gap in white noise
GS: recognition thresholds of temporally gated speech
ILD: interaural level difference
ITD: interaural time delay
NT: subjects without tinnitus
PTA: pure tone average
PTA_noise_: pure tone average in noise
PTAw: wide-range pure-tone average
PTAw_noise_: wide-range pure tone average in noise
SIN: speech recognition thresholds in competitive babble noise
SNR: signal to noise ratio
SRS: speech recognition threshold in silence
T: subjects with tinnitus
T1: tinnitus patients with the THI score “slight”
T2: tinnitus patients with the THI score worse than “slight”
THI: Tinnitus Handicap Inventory questionnaire.
